# Supplementary material for: Novel Wolbachia strains in Anopheles malaria vectors from Sub-Saharan Africa
Source: Wellcome Open Res. 2018 Nov 27;3:113. Originally published 2018 Sep 12. [Version 2] doi: 10.12688/wellcomeopenres.14765.2 (PMC6234743; doi:10.12688/wellcomeopenres.14765.2)
Supplement: Supplementary file 1 [file wellcomeopenres-3-16284-s0000.tgz › cedeaf91-df98-4265-bd26-22f5b2f56565_Supplementary_table_1.docx]

**Additional sample details and ITS2 GenBank accession numbers.** The location, species and sample code are shown in addition to the Wolbachia status and ITS2 accession number on Genbank.

| **Location** | **Species** | **Sample ID** | **Wolbachia status** | **ITS2 accession number** |
| --- | --- | --- | --- | --- |
| Guinea: Kissidougou | *Anopheles* sp. O/15 | GUI-KSK1 | W- | MH598414 |
| Guinea: Kissidougou | *Anopheles gambiae s.s.* | GUI-KSK2 | W- | MH598415 |
| Guinea: Faranah | *Anopheles nili* | GUI-FAR1 | W- | MH598416 |
| Guinea: Faranah | *Anopheles nili* | GUI-FAR2 | W- | MH598417 |
| Guinea: Kankan | *Anopheles* sp. unknown | GUI-KAN1 | W- | MH598418 |
| DRC: Lwiro | *Anopheles* sp. A/1 | DRC-LWI1 | W+ | MH598419 |
| DRC: Lwiro | *Anopheles* sp. A/1 | DRC-LWI2 | W+ | MH598420 |
| DRC: Lwiro | *Anopheles* sp. A/1 | DRC-LWI3 | W+ | MH598421 |
| DRC: Katana | *Anopheles* sp. A/1 | DRC-KAT1 | W+ | MH598422 |
| DRC: Katana | *Anopheles* sp. A/1 | DRC-KAT2 | W+ | MH598423 |
| DRC: Mikalayi | *Anopheles moucheti* | DRC-MIK1 | W+ | MH598424 |
| DRC: Kinshasa | *Anopheles gambiae s.s.* | DRC-KIN1 | W+ | MH598425 |
| DRC: Mikalayi | *Anopheles gambiae s.s.* | DRC-MIK2 | W+ | MH598426 |
| DRC: Kalemie | *Anopheles gambiae s.s.* | DRC-KAL1 | W+ | MH598427 |
| DRC: Kalemie | *Anopheles arabiensis* | DRC-KAL2 | W+ | MH598428 |
| DRC: Katana | *Anopheles gambiae s.s.* | DRC-KAT3 | W- | MH598429 |
| Ghana: Dogo | *Anopheles coluzzii* | GHA-DOG1 | W+ | MH598430 |
| Ghana: Dogo | *Anopheles coluzzii* | GHA-DOG2 | W+ | MH598431 |
| Ghana: Dogo | *Anopheles coluzzii* | GHA-DOG3 | W+ | MH598432 |
| Ghana: Dogo | *Anopheles coluzzii* | GHA-DOG4 | W- | MH598433 |
| Ghana: Dogo | *Anopheles coluzzii* | GHA-DOG5 | W- | MH598434 |
| Ghana: Dogo | *Anopheles melas* | GHA-DOG6 | W- | MH598435 |
| Uganda: Butemba | *Anopheles gambiae s.s.* | UGA-BUT1 | W- | MH598436 |
| Uganda: Butemba | *Anopheles gambiae s.s.* | UGA-BUT2 | W- | MH598437 |
| Uganda: Butemba | *Anopheles gambiae s.s.* | UGA-BUT3 | W- | MH598438 |
| Uganda: Butemba | *Anopheles gambiae s.s.* | UGA-BUT4 | W- | MH598439 |
| Uganda: Butemba | *Anopheles arabiensis* | UGA-BUT5 | W- | MH598440 |
| Madagascar: Antafia | *Anopheles gambiae s.s.* | MAD-ANT1 | W- | MH598441 |
| Madagascar: Antafia | *Anopheles gambiae s.s.* | MAD-ANT2 | W- | MH598442 |
| Madagascar: Antafia | *Anopheles gambiae s.s.* | MAD-ANT3 | W- | MH598443 |
| Madagascar: Ambomiharina | *Anopheles rufipes* | MAD-AMB1 | W- | MH598444 |
| Madagascar: Ambomiharina | *Anopheles rufipes* | MAD-AMB2 | W- | MH598445 |
