# Supplementary material for: Novel Wolbachia strains in Anopheles malaria vectors from Sub-Saharan Africa
Source: Wellcome Open Res. 2018 Nov 27;3:113. Originally published 2018 Sep 12. [Version 2] doi: 10.12688/wellcomeopenres.14765.2 (PMC6234743; doi:10.12688/wellcomeopenres.14765.2)
Supplement: Supplementary file 2 [file wellcomeopenres-3-16284-s0001.tgz › c69e4bcb-1280-416e-92cd-e642c6af69b6_Supplementary_table_2.docx]

***Wolbachia* 16S and wsp GenBank accession numbers.** Sample codes, *Wolbachia* strain names and Genbank accession numbers for Wolbachia 16S and wsp genes.

| **Sample ID** | **Strain** | **16S** | **wsp** |
| --- | --- | --- | --- |
| DRC-LWI1 | *w*AnsA | MH605275 | MH605281 |
| DRC-LWI2 | *w*AnsA | MH605276 | MH605282 |
| DRC-LWI3 | *w*AnsA | MH605277 | MH605283 |
| DRC-KAT1 | *w*AnsA | - | MH605284 |
| DRC-KAT2 | *w*AnsA(2) | - | MH605285 |
| DRC-MIK1 | *w*AnM | MH605278 | - |
| GHA-DOG1 | *w*Anga-Ghana | MH605279 | - |
| GHA-DOG2 | *w*Anga-Ghana | MH605280 | - |
