# Supplementary material for: Novel Wolbachia strains in Anopheles malaria vectors from Sub-Saharan Africa
Source: Wellcome Open Res. 2018 Nov 27;3:113. Originally published 2018 Sep 12. [Version 2] doi: 10.12688/wellcomeopenres.14765.2 (PMC6234743; doi:10.12688/wellcomeopenres.14765.2)
Supplement: Supplementary file 3 [file wellcomeopenres-3-16284-s0002.tgz › 1b01a5a3-080a-4407-b0a4-01510147e312_Supplementary_table_3.docx]

***Wolbachia* MLST gene GenBank accession numbers.** Sample codes, *Wolbachia* strain names and Wolbachia MLST gene sequence Genbank accession numbers.

| **Sample ID** | **Strain** | **gatB** | **coxA** | **hcpA** | **ftsZ** | **fbpA** |
| --- | --- | --- | --- | --- | --- | --- |
| DRC-LWI1 | *w*AnsA | MH605286 | MH605290 | MH605295 | MH605299 | MH605302 |
| DRC-KAT1 | *w*AnsA | MH605287 | MH605291 | - | - | MH605303 |
| DRC-KAT2 | *w*AnsA(2) | - | MH605292 | MH605296 | - | - |
| DRC-MIK1 | *w*AnM | MH605288 | MH605293 | MH605297 | MH605300 | MH605304 |
| GHA-DOG1 | *w*Anga-Ghana | MH605289 | MH605294 | - | MH605301 | MH605305 |
| GHA-DOG2 | *w*Anga-Ghana | - | - | MH605298 | - | - |
