# Supplementary material for: Novel Wolbachia strains in Anopheles malaria vectors from Sub-Saharan Africa
Source: Wellcome Open Res. 2018 Nov 27;3:113. Originally published 2018 Sep 12. [Version 2] doi: 10.12688/wellcomeopenres.14765.2 (PMC6234743; doi:10.12688/wellcomeopenres.14765.2)
Supplement: Supplementary file 4 [file wellcomeopenres-3-16284-s0003.tgz › 2f616f2c-3816-4b6b-806a-8278b81bbfef_Supplementary_figure_1.docx]

**Alpha and beta diversity of *An. gambiae* s.s. from Kissidougou, Guinea and Butemba, Uganda.** **A)** Alpha diversity using the Shannon diversity index shows the relative abundance of bacterial genera. **B)** To identify dissimilarities in the bacterial community structure between the microbiome, principal coordinates analysis (PCoA) was performed based on a Bray-Curtis dissimilarity matrix based on 97% clustered OTUs.
