# Supplementary material for: Novel Wolbachia strains in Anopheles malaria vectors from Sub-Saharan Africa
Source: Wellcome Open Res. 2018 Nov 27;3:113. Originally published 2018 Sep 12. [Version 2] doi: 10.12688/wellcomeopenres.14765.2 (PMC6234743; doi:10.12688/wellcomeopenres.14765.2)

**Prevalence of *Asaia* bacteria and malaria parasites in *An. gambiae* s.s. mosquitoes from Guinea**. **A)** Normalised *P. falciparum*: *An. gambiae* gene Ct ratio for mosquitoes that are infected with malaria and +/- *Asaia* bacteria. **B)** *P. falciparum* and *Asaia* infection rates (%) in 152 *An. gambiae* s.s. females. **C)** Box and whisker plot of Ct values for detection of *Asaia* and *P. falciparum* malaria showing more variable levels of *Asaia* detected.


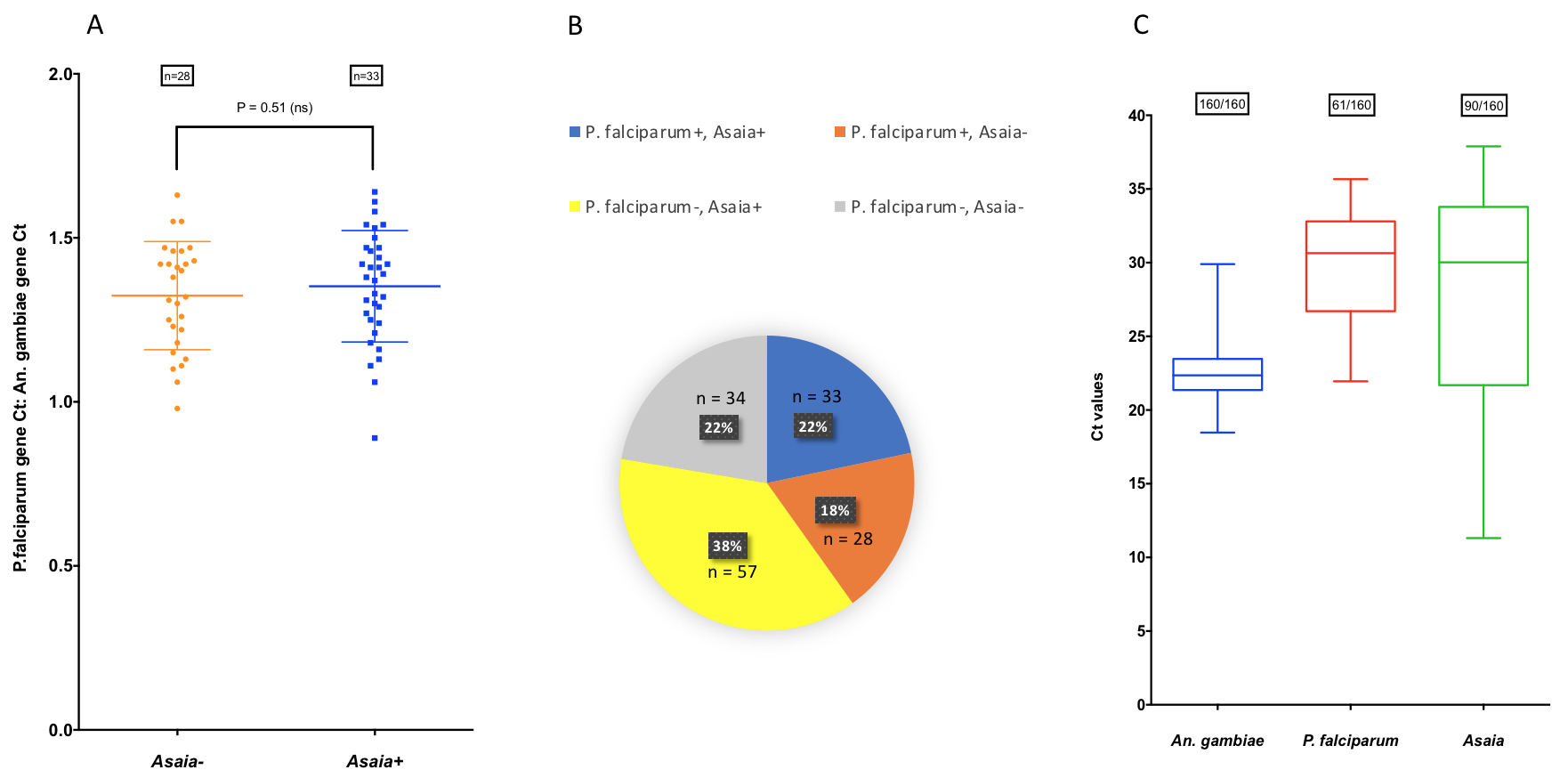

Supplement: Supplementary file 5 [file wellcomeopenres-3-16284-s0005.tgz › 6de9a49a-a713-4272-a714-465524623a89_Revised_supp_fig_2.docx]
