# Supplementary material for: Novel Wolbachia strains in Anopheles malaria vectors from Sub-Saharan Africa
Source: Wellcome Open Res. 2018 Nov 27;3:113. Originally published 2018 Sep 12. [Version 2] doi: 10.12688/wellcomeopenres.14765.2 (PMC6234743; doi:10.12688/wellcomeopenres.14765.2)
Supplement: Peer review history

# Peer review: Referee response for version 2

**DOI:** 10.21956/wellcomeopenres.16284.r34345

**Published:** 2018-12-11

**Authors:** Mathilde Gendrin, Ottavia Romoli

**Affiliations:** 1 Microbiota of Insect Vectors Group, Institut Pasteur de la Guyane, Cayenne, French Guiana; 2 Microbiota of Insect Vectors Group, Institut Pasteur de la Guyane, Cayenne, French Guiana; 3 Parasites and Vectors Department, Institut Pasteur, Paris, France

Our main comments have been addressed in the revised manuscript.

 We still don't understand what An. species A is (and even An species O). It is not referenced in Vectorbase, so is it just a species that the authors were not able to identify, or a recently identified species that we are not aware of? A short explanation on this would be helpful, especially that Wolbachia was specifically found at high prevalence in this mosquito species.

 Except for this small point, we think that the manuscript is sound and clear, and that conclusions are drawn adequately.

We have read this submission. We believe that we have an appropriate level of expertise to confirm that it is of an acceptable scientific standard.

# Peer review: Referee response for version 1

**DOI:** 10.21956/wellcomeopenres.16091.r33880

**Published:** 2018-11-08

**Authors:** Christophe Antonio-Nkondjio

**Affiliations:** 1 Malaria Research Laboratory, Organisation for Coordination in the Fight Against Endemic Diseases in Central Africa, Yaoundé, Cameroon

Good study by Jefferies *et al.* presenting the distribution of Wolbachia strains in anopheles species from different sub-Saharan Africa countries.

**General comment**

 In the method section the authors say DNA was extracted from whole mosquitoes or abdomen for their analysis. What are the chances that wolbachia infections cases reported in the paper could be due to parasites contain in the blood meal rather than true infection of mosquitoes?

**Methods**

**Study sites & collection methods**

- “Democratic Republic of the Congo” change to “Democratic Republic of Congo”
- Collection sites it will be interesting to indicate from the coordinates if it is Latitude North/South or longitude East/West the paper is also for non specialists in the domain.

**Figure 1: **

- B, C, D in the legend it is mentioned “P. falciparum prevalence” is it for human or mosquitoes please provide precision. (% Positive ???, % Negative ??)
- P. falciparum should be in italics. 
- Figure 1A: It should be interesting to indicate the names of study sites. The authors could labelled the sites by using number for sites for each country 1, 2,3 … then providing in the legend what 1 is placed for. 

 

**Figure 6: **legend not clear. 

**Figure 7 B:** The legend is not clear (can’t read anything).

I have read this submission. I believe that I have an appropriate level of expertise to confirm that it is of an acceptable scientific standard.

# Peer review: Referee response for version 1

**DOI:** 10.21956/wellcomeopenres.16091.r34140

**Published:** 2018-11-05

**Authors:** Anne Duplouy

**Affiliations:** 1 Lund University, Lund, Sweden

Jeffries and colleagues provide here a study of the prevalence and penetrance of *Wolbachia* infection in several species of *Anopheles *mosquitoes across several Sub-Saharian countries. To my knowledge this is the largest such study on the topics. This study adds important data on growing evidence that species of the genus *Anopheles* can host the infection, thus contrasting with previous reports suggesting that the bacterium was absent from these mosquitoes.

 Furthermore, the authors investigate the tripartite occurence between *Wolbachia*, the parasite *Plasmodium* and another symbiotic bacterium *Asaia*. The article supports previous studies suggesting niche competition between *Wolbachia* and *Asaia*, as none of the samples carry both bacteria. The study does not, however, provide field-based evidence that the presence of *Wolbachia *and/or *Asaia* in the mosquitoes would affect parasitism by *Plasmodium.*

 This research is timely. With the development of new pest control strategies using *Wolbachia* as a natural biological agent against the transmission of several vector-borne diseases in the field, it is important to have a comprehensive understanding of the diversity of the natural infections already present in the field, but also of the different factors that could affect the efficiency of such control programs. Including the presence of competing natural infection by *Asaia* bacteria for example.

 The study is well written and clear, with the sufficient information included to support future potential replication. I think this is a fine contribution to the current literature, I have only minor comments to the authors.

 It might be worth modifying the text in the abstract, and the introduction, to specify that the previous reports of *Wolbachia* in *Anopheles* were only from 2 *West-African* countries, while the current study is providing data from 5 countries across the Sub-Saharian region.

 Method:

 Please provide information on how the maps of figure 1 were generated. Did you need any approval/licenses for using these maps?

 Please provide information on collection permits, if any was needed from the different African countries.

 What is CDC standing for in the method section? ‘CDC-light trap’

 In the *Wolbachia* detection method section:

 Edit typo: ‘was used AS a positive control’

 Table 1: What is the rational for the authors to provide the information by countries rather than by species? Isn’t the most interesting point of the paper about the infection being reported in additional species of *Anopheles*?

 Figure 2: Explain the significance of the difference square/circle/triangle shapes and filled vs empty shapes? Also state in legend that the codes given are the Genbank Accession numbers.

 Figure 2: Where did you get the sequences from the *An. bwambae* and *An. quadriannulatus*? I think this info is missing from the method section.

*P. falciparum*, Wolbachia and Asaia prevalence section, paragraph 2:

 Does your analysis include the *P. falciparum* and *Wolbachia* infected specimens? Would it make any difference to remove the *Wolbachia*-infected specimens from the analysis?

 Discussion section, end of 4 ^th^ paragraph:

 ‘New’ strain in *An. Coluzzii* from Ghana. ‘New’ sounds like the infection is more recent than any other infection found in this mosquito species, which the results are not supporting. Would ‘unique’ or ‘different’ be good enough?

 Figure 7: Where is *Asaia* from Figure 7b? from the current picture it looks like *Asaia* is absent from those samples. Although the text states that the infection is not a dominant species of those samples. If *Asaia* is included in the ‘Others’ maybe it is worth specifying it in the legend, otherwise it could be added as a particular section of the graph like in Figure 7a to ease comparison of the two panels.

 Figure S1: Why are some of the circles slightly larger than others? Is it that different samples are overlapping?

I have read this submission. I believe that I have an appropriate level of expertise to confirm that it is of an acceptable scientific standard.

# Peer review: Referee response for version 1

**DOI:** 10.21956/wellcomeopenres.16091.r33884

**Published:** 2018-09-21

**Authors:** Mathilde Gendrin, Ottavia Romoli

**Affiliations:** 1 Microbiota of Insect Vectors Group, Institut Pasteur de la Guyane, Cayenne, French Guiana; 2 Parasites and Vectors Department, Institut Pasteur, Paris, France; 3 Microbiota of Insect Vectors Group, Institut Pasteur de la Guyane, Cayenne, French Guiana

Jeffries and co-authors performed a large scale analysis of the presence of Wolbachia in Anopheles mosquitoes from five countries in Africa. They found that in two of these countries, some mosquitoes were infected with Wolbachia, confirming and widening the recent discovery of the presence of Wolbachia in Anopheles mosquitoes. This is the strongest point of the paper, as an independent confirmation is always welcome and as some populations of Anopheles are even found here to have a high prevalence of Wolbachia.

 The authors also checked for the presence of Asaia sp. in the analysed mosquitoes, as this bacterium is thought to compete with Wolbachia in Anopheles. They did not find any mosquito co-infected by Asaia and Wolbachia. This is also an important finding as it corroborates studies performed in the laboratory, but this time with field-collected mosquitoes. They found that in mosquitoes coming from one population, Asaia was actually a dominant species, >99% of the microbiota. Figure 7a is not very clear as one expects the scale to go from 0 to 100%, therefore we suggest to use a discontinued axis to present these interesting results.

 Finally the authors investigated the presence of Plasmodium in the studied mosquitoes, as Wolbachia is thought to interfere with some transmitted pathogens. This part is less convincing as the tests have been performed on DNA extraction from whole bodies or abdomens, while the presence of Plasmodium in head and thorax (or more specifically, in salivary glands) is a more suitable method to assess transmission potential. Moreover, the conclusions drawn on the interactions between Plasmodium and Wolbachia are not exactly clear. Considering that 10.16 + 1.56 = 11.72% mosquitoes are infected with Wolbachia and 11.72 + 1.56 = 13.28% are infected with Plasmodium, if there is no effect between Wolbachia and Plasmodium, you expect that 11.72% x 13.28% = 1.56% is infected by both. Surprisingly, this is exactly the result here. Biology is rarely so close to math, for so small numbers… The authors should thus state more clearly that their results favor no interactions, as confirmed by the p value which is very close to 1. On the contrary, the discussion currently suggests that the non significant correlation is due to small numbers. However, one cannot jump to conclusion on the inability of Wolbachia to interfere with Plasmodium, as these results have been performed on abdomens and whole bodies, therefore we do not know whether the co-infected mosquitoes had just blood fed (and/or carried early stages of Plasmodium).

 To improve the clarity of the article, it would be interesting to have an additional figure or table summarizing the experimental set up, explaining which mosquitoes are included in which analysis and which Wolbachia strain is found in which mosquitoes.

 We also have minor comments on the manuscript:

 The expression « resident strain » is not clear to us.

 16S « rRNA » and « rDNA »: a consistent word may be used, rRNA seems more consensual.

 The total number of mosquitoes, of Wolbachia infected mosquitoes, of Asaia infected ones, etc would be interesting.

 Page 3:

 §2: Asaia is not an endosymbiont

 §3: « have » needs probably to be removed in « than would have be identified using morphological identification alone »

 §4 needs a first sentence identifying the gap of knowledge that the authors want to fill

 §5: Can the authors clearly state whether some mosquitoes had blood in their midgut?

 Page 4:

 Figure 1: scale should be in km, miles is not an SI unit

 §2: « DNA extraction and MOSQUITO species identification ». More generally, it is not always clear whether the authors speak about mosquitoes or Wolbachia strains.

 §3: « as preliminary trials revealed this was the optimal method for both sensitivity and specificity »: please add « data not shown » or remove it

 Page 5:

 Instead of µL of DNA, the actual quantity in ng would be preferable.

 All PCRs: primer sequences are needed

 §3: « Both chromatograms (forward and reverse traces) from each sample WERE manually »

 Pages 6-7

 Table 1: probably some mistakes, e.g. An. gambiae in Mikalayi: 11.8% corresponds neither to 1/16 nor to 2/16, so all the numbers should be checked. It would be appropriate to enter the actual numbers in brackets, and to indicate the co-prevalence of Wolbachia and Plasmodium. The legend should be grouped below or above the table and the explanation about mosquitoes in bold is unclear.

 In the text, numbers would be interesting rather than only proportions.

 « previously named M molecular form OF AN. GAMBIAE » (or remove it, as this precision may now be superfluous). On the contrary, « An. species A » is barely introduced, it would be interesting to mention something about this species and its identification (besides the quick explanation in the introduction).

 Page 13

 « Approximately 1000-fold higher », it is very much of an approximation (variable Ct values and potential variations in 16S copy number): it may be good to rephrase, mentioning that 1000 is an order of magnitude rather than approximately.

 §2: « An. moucheti (wAnM-infected) » comes at the 2nd occurrence of wAnM.

We have read this submission. We believe that we have an appropriate level of expertise to confirm that it is of an acceptable scientific standard, however we have significant reservations, as outlined above.

# Response

**Published:** 2018-11-13

**Authors:** Thomas Walker

**Affiliations:** London School of Hygiene & Tropical Medicine, UK

Dear Mathilde and Ottavia,

Firstly many thanks for your thoughtful and comprehensive review of our manuscript.  We have tried to address all your comments below in **bold**: 

Figure 7a is not very clear as one expects the scale to go from 0 to 100%, therefore we suggest to use a discontinued axis to present these interesting results.

**We agree and have modified this figure for clarity**

Finally the authors investigated the presence of Plasmodium in the studied mosquitoes, as Wolbachia is thought to interfere with some transmitted pathogens. This part is less convincing as the tests have been performed on DNA extraction from whole bodies or abdomens, while the presence of Plasmodium in head and thorax (or more specifically, in salivary glands) is a more suitable method to assess transmission potential. Moreover, the conclusions drawn on the interactions between Plasmodium and Wolbachia are not exactly clear. Considering that 10.16 + 1.56 = 11.72% mosquitoes are infected with Wolbachia and 11.72 + 1.56 = 13.28% are infected with Plasmodium, if there is no effect between Wolbachia and Plasmodium, you expect that 11.72% x 13.28% = 1.56% is infected by both. Surprisingly, this is exactly the result here. Biology is rarely so close to math, for so small numbers… The authors should thus state more clearly that their results favor no interactions, as confirmed by the p value which is very close to 1. On the contrary, the discussion currently suggests that the non significant correlation is due to small numbers. However, one cannot jump to conclusion on the inability of Wolbachia to interfere with Plasmodium, as these results have been performed on abdomens and whole bodies, therefore we do not know whether the co-infected mosquitoes had just blood fed (and/or carried early stages of Plasmodium)

**We agree and have modified our discussion on these results to make more appropriate conclusions based on our data**

To improve the clarity of the article, it would be interesting to have an additional figure or table summarizing the experimental set up, explaining which mosquitoes are included in which analysis and which Wolbachia strain is found in which mosquitoes.

**Many thanks for this suggestion.  After careful consideration, we feel that an additional figure or table is not needed given we have figure 1 showing which *Anopheles*species were *Wolbachia*-infected and from which locations within countries and have all the PCR screening data from all samples available from** **Open Science Framework:  DOI 10.17605/OSF.IO/MW6XZ in addition to sample details for all accession numbers in the supplementary tables.**

**However, we have also modified table 1 to provide the comparison between Plasmodium-infected, Wolbachia-infected, Asaia-infected, co-infected individuals and uninfected individuals across all collection sites.**

We also have minor comments on the manuscript:

The expression « resident strain » is not clear to us.

**‘Resident’ *Wolbachia *strains are considered to have resulted naturally and have an evolutionary association with the host (wAlbA and wAlbB in Ae. albopictus) rather than have been generated artificially through transinfection (eg. wMel in Ae. aegypti).**

**We have modified our introduction to make this clearer by the inclusion of ‘those naturally present in wild insect populations’**

16S « rRNA » and « rDNA »: a consistent word may be used, rRNA seems more consensual.

**We agree with this.  For Wolbachia screening and phylogeny including strains in Anopheles, 16S rRNA is most commonly used (de Oliveira** **et al. 2015: Werren & Windsor 2000; Gomes et al. 2017; Baldini et al. 2014).** **We have checked our manuscript and corrected these errors.**

The total number of mosquitoes, of Wolbachia infected mosquitoes, of Asaia infected ones, etc would be interesting.

**We agree and have modified table 1 to include the number of infected mosquitoes for all categories (including uninfected individuals).**

Page 3:

§2: Asaia is not an endosymbiont

**We agree and have modified throughout the manuscript to reflect this mistake**

§3: « have » needs probably to be removed in « than would have be identified using morphological identification alone »

**We agree have corrected this sentence**

§4 needs a first sentence identifying the gap of knowledge that the authors want to fill

**We agree and have added the following sentence:  “** **Investigating the prevalence and diversity of *Wolbachia *strains naturally present in *Anopheles *populations across diverse malaria endemic countries would allow a greater understanding of how this bacterium could be influencing malaria transmission in field populations and provide candidate strains for transinfection” ** 

§5: Can the authors clearly state whether some mosquitoes had blood in their midgut?

**We did not fully determine the Sella score of the mosquitoes used in our study so our collection likely contained individuals that had undigested blood. However, we have the following sentences in our discussion which we feel acknowledges the limitations of our study:**

**“However, detection of *P. falciparum *parasites in whole body mosquitoes does not confirm that the species plays a significant role in transmission. Detection could represent infected bloodmeal stages or oocysts present in the midgut wall so further studies are warranted to determine this species ability to transmit human malaria parasites.”**

Page 4:

Figure 1: scale should be in km, miles is not an SI unit

**We have changed this to km**

§2: « DNA extraction and MOSQUITO species identification ». More generally, it is not always clear whether the authors speak about mosquitoes or Wolbachia strains.

**We have added the word ‘mosquito’ prior to species identification for clarity**

§3: « as preliminary trials revealed this was the optimal method for both sensitivity and specificity »: please add « data not shown » or remove it

**We have removed this as it’s been shown before in multiple previous publications and is a well-established PCR assay for detection of *Plasmodium*.**

Page 5:

Instead of µL of DNA, the actual quantity in ng would be preferable.

**Although we did measure total DNA for selected samples and normalised A** ***n. gambiae*** **extracts to Ct values for a single copy *An. gambiae rps17 *housekeeping gene, we did not do this for all species across all countries so for consistency we feel ul of DNA is more representative of our work**

All PCRs: primer sequences are needed

**We have added all primer sequences were appropriate**

§3: « Both chromatograms (forward and reverse traces) from each sample WERE manually »

**We have changed this grammatical error** 

Pages 6-7

Table 1: probably some mistakes, e.g. An. gambiae in Mikalayi: 11.8% corresponds neither to 1/16 nor to 2/16, so all the numbers should be checked. It would be appropriate to enter the actual numbers in brackets, and to indicate the co-prevalence of Wolbachia and Plasmodium. The legend should be grouped below or above the table and the explanation about mosquitoes in bold is unclear.

In the text, numbers would be interesting rather than only proportions.

« previously named M molecular form OF AN. GAMBIAE » (or remove it, as this precision may now be superfluous). On the contrary, « An. species A » is barely introduced, it would be interesting to mention something about this species and its identification (besides the quick explanation in the introduction).

**We have modified table 1 for clarity including numbers and removed the reference to M and S forms. The legend format is according to WOR guidelines and we have modified the table legend for clarity.**

**As very little is known about An. species A and what we were able to find on this species is presented in our discussion “** ***An.*** **species A should be further investigated to determine if this species is a potential malaria vector, given our study demonstrated** ***P. falciparum*** **infection in one of two individuals screened and ELISA-positive samples of this species were reported from the Western Highlands of Kenya** **^42^** **.”**

Page 13

« Approximately 1000-fold higher », it is very much of an approximation (variable Ct values and potential variations in 16S copy number): it may be good to rephrase, mentioning that 1000 is an order of magnitude rather than approximately.

**We have modified this sentence**

§2: « An. moucheti (wAnM-infected) » comes at the 2nd occurrence of wAnM.

**We have corrected this by removing ‘An. moucheti’**
